# Biology, Buddhism, and AI: Care as the Driver of Intelligence

**DOI:** 10.3390/e24050710

**Published:** 2022-05-16

**Authors:** Thomas Doctor, Olaf Witkowski, Elizaveta Solomonova, Bill Duane, Michael Levin

**Affiliations:** 1 Centre for Buddhist Studies, Rangjung Yeshe Institute, Kathmandu University, Kathmandu 44600, Nepal; thomas.doctor@ryi.org (T.D.); public@billduane.com (B.D.); 2 Center for the Study of Apparent Selves, Rangjung Yeshe Institute, Kathmandu 44600, Nepal; olaf@cross-compass.com (O.W.); elizaveta.solomonova@mcgill.ca (E.S.); 3 Cross Labs, Cross Compass Ltd., Kyoto 604-8206, Japan; 4 College of Arts and Sciences, University of Tokyo, Tokyo 113-8654, Japan; 5 Earth-Life Science Institute, Tokyo Institute of Technology, Tokyo 145-0061, Japan; 6 Neurophilosophy Lab, Department of Psychiatry, McGill University, Montreal, QC H3A 0G4, Canada; 7 Bill Duane and Associates LLC, San Francisco, CA 94117, USA; 8 Allen Discovery Center, Tufts University, Medford, MA 02155, USA; 9 Wyss Institute for Biologically Inspired Engineering, Harvard University, Boston, MA 02115, USA

**Keywords:** computer science, basal cognition, Buddhism, compassion, bioengineering, intelligence, stress, AI, embodiment, artificial life

## Abstract

Intelligence is a central feature of human beings’ primary and interpersonal experience. Understanding how intelligence originated and scaled during evolution is a key challenge for modern biology. Some of the most important approaches to understanding intelligence are the ongoing efforts to build new intelligences in computer science (AI) and bioengineering. However, progress has been stymied by a lack of multidisciplinary consensus on what is central about intelligence regardless of the details of its material composition or origin (evolved vs. engineered). We show that Buddhist concepts offer a unique perspective and facilitate a consilience of biology, cognitive science, and computer science toward understanding intelligence in truly diverse embodiments. In coming decades, chimeric and bioengineering technologies will produce a wide variety of novel beings that look nothing like familiar natural life forms; how shall we gauge their moral responsibility and our own moral obligations toward them, without the familiar touchstones of standard evolved forms as comparison? Such decisions cannot be based on what the agent is made of or how much design vs. natural evolution was involved in their origin. We propose that the scope of our potential relationship with, and so also our moral duty toward, any being can be considered in the light of Care—a robust, practical, and dynamic lynchpin that formalizes the concepts of goal-directedness, stress, and the scaling of intelligence; it provides a rubric that, unlike other current concepts, is likely to not only survive but thrive in the coming advances of AI and bioengineering. We review relevant concepts in basal cognition and Buddhist thought, focusing on the size of an agent’s goal space (its cognitive light cone) as an invariant that tightly links intelligence and compassion. Implications range across interpersonal psychology, regenerative medicine, and machine learning. The Bodhisattva’s vow (“for the sake of all sentient life, I shall achieve awakening”) is a practical design principle for advancing intelligence in our novel creations and in ourselves.

## 1. Introduction

The fields of basal cognition, Buddhist philosophy, computer science, and cognitive science are all concerned with fundamental questions around intelligence. What is unique about certain configurations of matter that enable them to exhibit intelligent behavior? How do the kinds and degrees of intelligence differ across beings? What processes drive the expansion of intelligence on evolutionary time scales, and what causes changes in the intelligence of a being during its lifespan? How can we understand intelligence in a way that would enable us to create novel instances, as well as improve our own intelligence for life-positive outcomes for all? Traditional approaches to this question have been focused on a set of standard “model systems” such as human subjects and certain animals (rats, birds, etc.) in the context of a historical evolutionary lineage on Earth. However, recent approaches in artificial intelligence and synthetic bioengineering have begun to produce novel types of agents whose intelligence cannot be readily predicted from the details of their construction or their origins [1,2,3,4]. These constructivist efforts to create intelligence in novel implementations (ranging from novel combinations of engineered living tissue to software) reveal key gaps in our understanding of dynamic intelligence [5]. Given the inevitable developments in the biological sciences, and the profound challenges faced by society, it is essential to develop frameworks that help us to detect, understand, and communicate with intelligences in unfamiliar guises. Here, we propose that Buddhist thought, and its emphasis on care and compassion as a catalyst of positive change in intelligent beings, is an empirically fruitful lens with which to understand intelligence.

A few words on methodology are perhaps in order here. This exploration across otherwise typically disparate scientific disciplines and scholarly contexts aims at achieving what can be thought of as “deep integration” [6]. Such an integrational approach does not privilege the discourse of any one particular discipline as the primary carrier of meaning into which statements and insights derived from other, complimentary frameworks and approaches must be translated. Instead, what we here seek to achieve is a form of mutually informed, explorative conversation that engages our customary, discipline-specific frameworks on equal footing, thereby facilitating the recognition of individual blind spots as well as otherwise unacknowledged, shared concerns. As a consequence of taking this approach, certain concepts that are central to this paper—such as stress, care, intelligence, self, or agency—take on a significance that emerges within and is defined by the concrete interdisciplinary encounter. For this reason, we have supplied a Glossary that explains a selection of such broadly applicable concepts as they are understood in the specific context of this paper. Perhaps in line with age-old Buddhist sentiments, our concern is here pragmatic before philosophical. At the same time, we hope that any instances of functional, conceptual integration that this paper may achieve can in turn motivate refinements and constructive research across the sciences of life, cognition, and information, as well as indeed in fields such as philosophy and religion. To illustrate this with an example, one of the main points of our definition of Self (as given in the Glossary and extracted from previous research) is that a Self is an illusory modelling construct created by perceptual systems of Agents. Agents construct models of causal Selves for others, and for ourselves, using the same machinery. The same mechanisms that cause an agent to act toward stress reduction in itself (even though the beneficiary of those actions is in an important sense impermanent) can be expanded to extend to other Selves. In this way, while our focus is on understanding and formulating Self in a way that is applicable to a broad range of scientific contexts, we also see ourselves as here contributing to the treatment of perennial issues in contemporary Buddhist philosophy—such as the feasibility of genuine care in a world without real individuals [7,8,9,10,11,12,13]. Similarly, with respect to the paper’s main thesis regarding care as a driver for intelligence: we hope that apart from addressing contemporary scientific or social aims and practices, our discussion may as well contribute to an understanding of classic Buddhist doctrine in its own right.

The field of basal cognition [14,15,16,17,18] emphasizes a continuum of intelligence, which originated in the control loops of microbes but was scaled up throughout multicellular forms to the obvious kinds of intelligent behavior observed in advanced animals. The emphasis on functional problem-solving, learning, and creative responses to challenges enables a focus on the central invariant of intelligence, not contingent facts and frozen accidents of the evolutionary journey of life on Earth. Given that intelligent behavior does not require traditional brains [16,18], and can take place in many spaces besides the familiar 3D space of motile behavior (e.g., physiological, metabolic, anatomical, and other kinds of problem spaces), how can we develop rigorous formalisms for recognizing, designing, and relating to truly diverse intelligences?

One way to think about a general, substrate-independent definition of “Intelligence” is centered on goal-directed activity [19,20]: what is common to all intelligent systems, regardless of their composition or origin, is the ability to display a degree of competency in reaching a goal (in some problem space, [21]) despite changing circumstances and novel perturbations. These ideas extend classical discussions by Spinoza, Kant, Jonas, and Heidegger. All intelligences, no matter how embodied, can be compared directly with respect to the maximum spatiotemporal scale of the goals towards which they can represent and work. A corollary to this view is that the driver of this kind of homeostatic dynamic is that such systems exhibit “stress” (the delta between current state and optimal state, or the difference between the goals at different subsystems’ levels): reduction of this stress parameter is a driver that keeps the system exerting energy in action to move and navigate within the problem space. It should be noted that stress can be seen as the inverse of “satisfaction” [22], and is relative to a contextual and non-stationary target.

**Figure 1:**
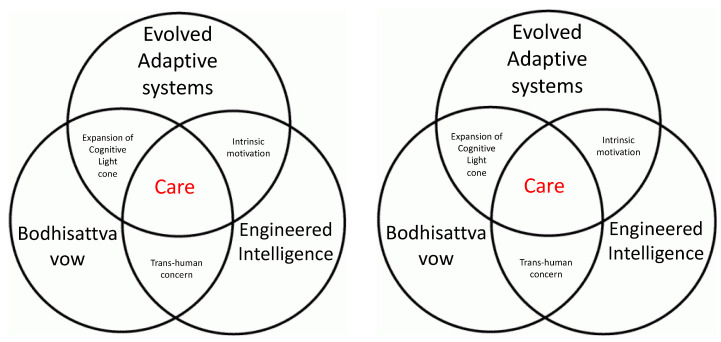
Care as the central invariant of a new interdisciplinary field. Legend: A schematic mindmap showing how Care is a central invariant binding across several fields. Care, or the capacity to exert energy and effort toward preferred states, is a central concept in Biology (because of the ubiquity of homeostatic loops at various scales of organization of life), in AI (because of the necessity to specify objective functions for artificial agents to follow), and Buddhism (because of the centrality of the concept of concern for all sentient beings’ welfare and progress). A commitment to maximization and scaling of outward-facing Care with respect to other agents’ goals, as occurs during the Bodhisattva vow, is a powerful driver concept for progress in synthetic and natural evolution (via scaling of goals from metabolic scalars to patterns of anatomical complexity in morphospace and eventually to complex behaviors in 3D space) and in AI (via a focus on building synthetic systems with the capacity to increase and modify their own cognitive boundaries).

Evolution enables the scaling of intelligence by exploiting biophysical mechanisms that enable progressively larger goal states (and thus progressively more complex causes of stress) to be represented and pursued [23]. More complex and advanced cognitive agents are capable of being stressed by larger and more complex states of affairs [24], regardless of their specific composition or provenance. These ideas are novel and somewhat disruptive for many traditional approaches that have been largely focused on brains and do not comfortably stretch to encompass advances in bioengineering, chimeric technologies, and machine learning. In complement to the Western traditions that have driven now-dissolving boundaries between brain, body, and environment [25], we propose that Buddhism offers an approach that is uniquely suited to the new field developing at the intersection of computer science, bioengineering, and cognitive science (Figure 1).

We propose a central concept as a key invariant across these fields: Care (a metric focused on motivation, stress, and goal-directedness of agents). If stress is the manifest discrepancy between current and optimal conditions, “Care” can in turn be defined as concern for stress relief, and “intelligence” as the degree of capacity for identifying and seeking such relief. By analyzing the role of Care in diverse contexts, informed by a Buddhist approach, we propose a new path towards improving both natural and artificial intelligence via a commitment to radical expansion of a being’s or an agent’s cognitive boundary: the scale of the things it can possibly care about (defined by the range of states that cause it stress and cause it to exert effort to change). In this framework, what an agent can possibly care about is a central determinant of its degree of intelligence. Importantly, this view not only helps us understand the origins and implementation of diverse types of intelligence within an agent, but also helps clarify the changes of an agent’s intelligence in its outward-facing relationships to other agents.

Whereas the drive to reduce one’s own stress is a primitive and universal ingredient in cognition and intelligence, the inclusion of others’ stress as a primary goal necessarily increases the cognitive boundary of an individual and scales its intelligence. Given the modular nature of homeostatic loops, this only requires that sensors that normally gauge the agent’s own states (face inwards) expand to include information about others’ states (start to face outwards). In this framework, the recognition of agency outside oneself and the progressive inclusion of their states in one’s own homeostatic stress-reduction loops is a bidirectional feedback loop that leads to the scaling of intelligence and increases in practical compassion. This loop operates on both the evolutionary and individual lifespan time scales, and in more advanced forms, comes under rational control of systems whose primary goals may start to include the meta-cognitive goal of increasing intelligence and compassion.

Advanced intelligence includes the ability to notice agency, and thus stress, and to seek its reduction. We employ this perspective on intelligence in an analysis of the Bodhisattva principle of agency and cognition, focusing on the traditional concept of “taking the Bodhisattva vow” and so committing to the pursuit of cognitive perfection (“awakening,” Skt. *bodhi*) for the benefit of all sentient beings throughout time and space [26,27]. In addition to better ways to understand biology, this framework suggests a number of conclusions with respect to stress transfer and goal identification that can serve as design principles for improved general artificial intelligence systems.

## 2. The Cognitive Light Cone Framework: Cognitive Boundaries, Goal-Directedness, and Domains of Concern

Many definitions of intelligence and cognitive capacity have been debated over the centuries [28]. The problem with most existing formalisms is that they are closely tied to a specific type of subject—such as humans, rats, birds, etc.—a traditional animal at a single “privileged” size and temporal scale [29], or even type of anatomy. Comparing intelligences among different animals, such as octopuses and dogs, is very challenging because their diverse environments and behaviors underline the fact that intelligence can be hard to ascertain in unfamiliar guises. An even bigger limitation in this field is the impending explosion in the prevalence of truly unusual living creatures and distributed systems [30]. Novel living beings produced by engineering and hybrid approaches (such as evolutionary design) include ex vivo constructs such as embryoids, organoids, and assembloids [31,32], cyborgs of animals and plants [3,4,33,34,35,36,37,38] resulting from living tissue tightly integrated with designed inorganic interfaces [39,40] and with closed-loop control systems [41], biological robots such as computer-controlled invertebrates [42,43,44], and hybrots consisting of living brains instrumentized to control artificial new bodies [1,43,45,46,47,48,49]. Without any familiar phylogenetic guideposts (e.g., “it’s a kind of fish so we expect a fish-like range of behaviors”), it may be extremely difficult to place the intelligence of novel, synthetic creatures with respect to the familiar examples. Recent efforts in “Diverse Intelligences” and Artificial Life initiatives seek to acknowledge the wide range of “life as it can be” [50,51], and produce frameworks for understanding of intelligence that not only subsume possible living beings (designed and evolved) but also include potential exobiological intelligences and purely software (AI) creations. The quest to be able to directly compare truly diverse intelligences, regardless of their origin story or composition, requires us to be able to specify the most general invariant underlying intelligence and cognition: what do all cognitive agents, no matter how advanced or humble, have in common?

One such framework has been suggested, and is focused on a candidate for an invariant that enables direct comparison of truly diverse agents (regardless of their composition or origin) [23,24,52]: goal-directedness. We suggest that an essential nature of cognition, in any embodiment, is the capacity for goal-directed activity in some problem space. In this sense, goal-directedness does not require a high-level self-awareness, but only a cybernetic kind of teleonomic functionality [53,54,55,56,57,58,59,60] (of course, other frameworks could be developed around different invariants such as information processing [61]).

**Figure 2:**
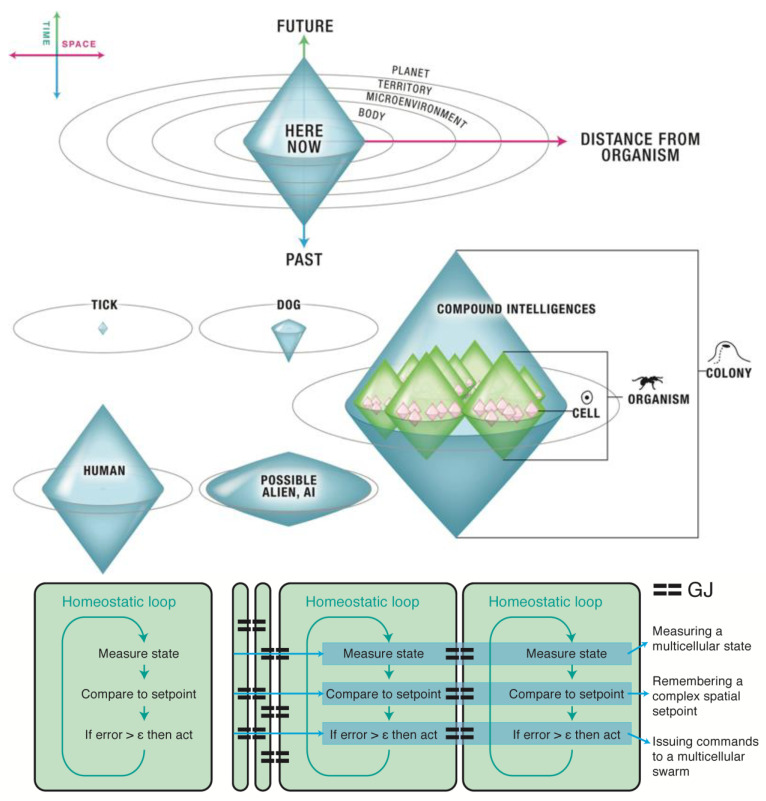
Legend: A focus on the size or scale of goals any given system can pursue, as an invariant across the space of possible sentient beings of whatever embodiment, allows plotting very diverse intelligences on the same graph [23]. The scale of their goal-directed activity is estimated (collapsed onto one axis of space and one of time, as in space-time diagrams). Importantly, this way of visualizing the sophistication of agency is a schematic of goal space—it is not meant to represent the spatial extent of sensing or effector range, but rather the scale of events about which they care and the boundary of states that they can possibly represent or work to change. This defines a kind of cognitive light cone (a boundary to any agent’s area of concern); the largest area represents the “now”, with fading efficacy both backward (accessing past events with decreasing reliability) and forward (limited prediction accuracy for future events). The diamond or “spinning top” shapes of the cones depicted above are simplifications. Agents are compound entities, composed of (and comprising) other sub- or super-agents, each of which has its own cognitive boundary of various sizes. Image by Jeremy Guay of Peregrine Creative. Selves increase their cognitive boundary by connecting together (“GJ”, standing for gap junctions—an example of a biophysical connection used by cells to merge into higher-level beings) in functional ways that allow simple homeostatic loops to measure, implement, and remember progressively larger states (thus increasing the scale and complexity of what they Care about). Top panel used by permission, produced by Jeremy Guay of Peregrine Creative.

On this view, any possible agent can be represented by drawing the spatiotemporal boundaries of the biggest goals which it is capable of pursuing (Figure 2). Tell me what you care about—what you actively spend energy on trying to achieve despite perturbations and novel situations—and I can immediately gauge your degree of sophistication. A bacterium can try to manage local sugar concentrations, with a bit of memory and a bit of predictive power. A dog has a larger area of concern, significant memory and predictive capacity in the short term, but it is probably impossible for it to care about something that will happen 100 miles away, 2 months from now. Humans have a huge cognitive envelope, perhaps uniquely one that is larger than our own lifespan; our state of being a creature capable of goals that appear fundamentally unachievable is very characteristic of Buddhist practice. Every creature thus has a “cognitive boundary”—which can be represented in the form of a light cone within space and time that demarcates the edge of what it can care about (not the first-order boundary of what it can sense and affect, but the second-order boundary demarcating the scale of its possible goals).

Analyzing systems with respect to this aspect has several advantages. The first is that it is completely agnostic about the composition of agents, enabling the most basal, primitive forms to be compared on the same scale as humans (whose cognitive boundary might extend to planetary scales) and novel life forms (synthetic, exobiological, etc.). Another advantage is that the continuous space underscores the futility of binary categories (“is it intelligent?”, “is it cognitive?”, “is it a real decision or just physics?”): modern bioscience offers no support for some sort of clean bright line separating cognitive beings from non-cognitive ones. All of the interesting capacities of mind, like the ones of the body, evolved gradually. Taking evolution seriously means asking “what kind” and “how much”, with respect to intelligence and cognition broadly conceived [62]. This is consistent not only with the facts of bioengineering (that any purportedly “non-cognitive” system can be mixed and hybridized with a cognitive one), but also with the evolutionary history of cognition. All of the main components of neurons (ion channels, electric synapses, neurotransmitter machinery, etc.) existed long before brains appeared—they were present in our unicellular ancestors. Indeed, evolution long ago (about the time of bacterial biofilms [63]) discovered that bioelectric networks are an ideal medium for scaling computation, coordinating and synthesizing information across distance, and implementing memory and reprogrammability. Developmental bioelectricity [64] is the medium by which non-neural cells form networks to manage morphogenesis in development and regeneration. Pre-neural bioelectric networks in the body underlie large-scale anatomical decision-making and possess instructive pattern memories that guide growth and form [64,65]. It is very likely that this system served as a precursor to neurobiology: prior to electrical networks controlling muscles to move the body through 3D space, these same networks generated signals to control cell behaviors in the body to move the body configuration through anatomical morphospace. Thus, anatomical homeostasis is a goal-seeking capacity of the collective intelligence of cellular swarms comprising living bodies [24].

## 3. Two Distinct Light Cones: One for Physical States, One for Care

**Figure 3:**
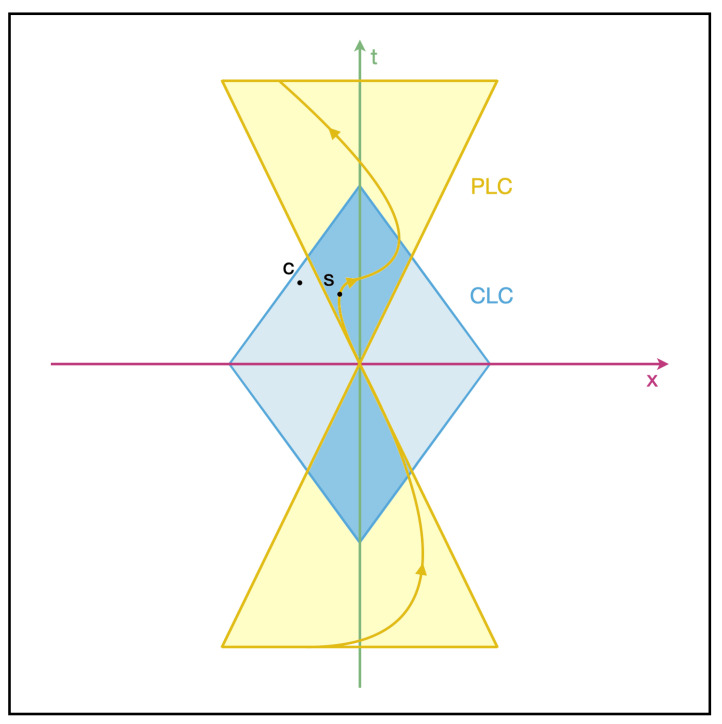
An agent’s boundary of Care. Legend: An agent’s Care light cone represented at a given time (CLC, in blue) and the corresponding agent’s physical light cone (PLC, in yellow). The diagram depicts a space where each point corresponds to a state of the agent. The agent cares for a state *s*, which is achievable, whereas it cares also for a state *c*, which is not reachable from the here and now (the present state is at the origin of the plot). Points that are outside CLC and PLC are respectively too distant in space or time to have any interaction with the agent, or too distant to be cared for by the agent.

According to the goal-directed model of intelligent agents, any individual agent is then delimited by the spatiotemporal boundary of events that it can seek to measure, model, and affect [23]. This surface sets a functional boundary, or “light cone” of its cognitive ability. We have considered the way systems may exchange stress between each other by exchanging signals, which has direct translations into the world of machine learning agents as well as natural agents. Agents may progressively come to reduce their levels of stress and transfer them between each other in more efficient ways, by means of communicating their goals. It may be helpful to clarify the difference between the goal-defined light cone (Figure 2) and a mere behavioral space light cone. While the latter merely defines the space of possible states in which an agent can find itself (defined by its position, speed, temperature, etc.), the light cone we defined above rather characterizes the maximum extent of the goals and aspirations of an agent, or in other words, its capacity for Care. An agent’s Care light cone (CLC) and its corresponding physical light cone (PLC) of behavioral space can be brought together in the same representation (Figure 3). In our representation of light cones, the two diagonal lines represent the two extrema in terms of physical change of the system state, while the horizontal line indicates the present state space. Anything outside of the cones that is located in the future cannot be reached from the present state in the future, nor can anything in the past that lies outside the cones be influencing the present state.

Light cones may be represented in two dimensions as in Figure 3. In our depiction of light cones, both physical states and cared-for states belong to their respective light cones. The diagram symbolically shows a phase space where each point corresponds to a state. Points within the Care light cone (CLC, represented in blue) represent states for which the agent cares at a given point in time—typically the present time in our depiction, but one may picture a time series of such light cones changing in time—rather than the states the agents are physically in. A state may be situated within a Care light cone even if it is too distant in space or time to have any interaction with the agent. Conversely, some states in the physical light cone (PLC, represented in yellow) may be beyond the light cone of Care of the agent, yet remain physically achievable through a certain trajectory. Goals cease to exist as soon as the self (the Care light cone, in blue) is reduced to a point, or, on the contrary, extends infinitely over the whole phase space of possible states (in yellow).

The light cones we consider are clouds of possibilities for agents, meaning that they represent distributions of probabilities of Care and physical states achievable in time and space. We note that the two types of light cones we describe naturally take two different shapes. On the one hand, the physical light cone determines the limited subspace corresponding to an initial state of the world undergoing change in accordance with a set of physical laws, similar to the light cones in the theory of relativity or light cones of information in evolutionary theory [66]. On the other hand, Care light cones need not bear such a limitation since an agent may care about entities that are not within reach in space and time. A light cone does not exist in a vacuum. On the contrary, it corresponds to a given substrate, and is surrounded by other light cones so that it may even overlap or contain other ones. The act of extending a light cone bears some connection with the act of several agents cooperating or acting as a cognitive whole as their Care light cones may start overlapping.

## 4. Problem Space, Fields of Stress, and Continuity of Cognitive Forms of Life

In this framework, intelligence is the degree of sophistication an agent can muster in navigating some specific problem space. Defined very broadly, problem spaces can be seen as fields that emerge in the gap between current and optimal conditions—or in other words, as fields of stress. The generalization of problem spaces beyond the traditional 3D space of “behavior” into other, virtual problem spaces is essential for understanding evolution of basal cognition. Living things first solved problems in metabolic space, and evolution then pivoted the same kinds of strategies to solve problems in physiological, transcriptional, and anatomical space, before speed-optimizing these dynamics to enable rapid behavior in 3D space. Since every cognitive agent is made of parts, it is essential to have a theory about how numerous goal-seeking agents link together into a new, larger cognitive system that is novel and not present in any of the subunits. The multiscale competency architecture of life [24] is such a hypothesis about the scaling of cognition, seeing complex system-level behaviors in any space as the within- and across-level competition and cooperation among the various subunits and partitions of composite agents (i.e., all agents).

This emphasis on the fundamental continuity, kinship, and infinite variety of life and cognition resonates with Buddhist descriptions of continuous cycles of life and death that emerge through infinite causal dependencies and with potential for radical bodily and cognitive transformation [26,67]. Another feature of this vision that aligns well with Buddhist ideas is the lack of a permanent, unique, unitary Self [68]. The picture given by the evolutionary cell-biological perspective is one where a cognitive agent is seen as a self-reinforcing process (the homeostatic loop), not a thing [69,70,71]. Of course, on long time scales, *all* objects are, consistent with Mahāyāna Buddhist perspectives, just temporary configurations—the distinction between permanent objects and temporary bundles of coherent processes (cf. the Ship of Theseus paradox and Nāgasena’s chariot parable as a description of biological beings [72,73]) fades away.

In this society of processes, overlapping goal-seeking partitions are all interacting with each other. Importantly, the boundaries of these Selves not only interpenetrate, but can also change during the agent’s lifetime. Defections from large-scale anatomical goals, such as those that occur due to an inappropriate reduction of gap junctional connectivity [74], present as cancer, cause reversions of cell behavior to ancient unicellular concerns which lead to metastasis and over-proliferation as the cells treat the rest of the body as external environment.

Another key fundamental commonality is the focus on striving. The central treadmill (loop) of life is a homeostatic effort to attain a specific setpoint, despite the buffeting influence of the cruel, dissipative environment. The driver of this loop is stress—the delta between current state and desired state, and all of the system’s efforts are guided by the effort to minimize stress (essentially, unhappiness). Defined in this way, “stress” turns out to be a compelling translation of the Sanskrit term *duḥkha* (otherwise often rendered as “suffering”), which describes a treacherous world inhabited by restlessly craving beings. In this world of stress, existence equals dissatisfaction, and so *duḥkha* is a continuous state that compels beings to act [67]. This stress-focused perspective can be seen as suggesting that the expansion of cognition across eons was basically a process of scaling goals, from humble metabolic needs to single cells to the grandiose goals of “make and maintain a whole limb” of tissue- and organ-level cellular collectives. It is fascinating to think about how this expansion of concern scales basic self-preservation goals into outward-facing preferences about complex, large states of the environment and even care for the states of other beings.

One instructive example is what happens in bioelectric networks during multicellularity. Cells join into networks with electrical synapses known as gap junctions. What is special about these is that unlike traditional signaling (by diffusible secreted chemical signals and receptors), information molecules pass through gap junctions *directly into the internal milieu of the recipient cell.* Once the signal is inside a cell, that cell cannot tell whether this molecule is a memory trace of something that happened to that cell (a true memory engram) or a false memory incepted into its informational structure by a kind of memory transfer from its neighbor. Imagine for example a calcium spike due to an injurious stimulus: calcium has no metadata on it to describe whose signal it is, and once it spreads across a few cells, they become a collective that has information about injury that is distributed across the individuals. In effect, it performs a kind of “mind meld”, binding subunits into a collective because it becomes very difficult to keep individualization regarding which cell has which information. It is hard to maintain the I-you distinction, and cooperation is massively favored. This is not because the agents have become less selfish, but because the size of the self (to which they are committed) has grown. For properly coupled cells, it is impossible to hide information from each other (from yourself) and it is impossible to do anything injurious to your neighbor because the same effects (consequences) will affect you within seconds. Gap junctions provide an efficient life-transforming dynamic—cause and effect which massively augments cooperative interactions. The eventual result is the scaling up of the cognitive boundary, the processing capacity, the information content and structure, and the goals. An individual cell strives to become two cells. A gap-junction-coupled collective strives to make an organ, being able to represent goal states such as number of fingers which are unfathomable to individual cells. In connecting with others in a strong informational sense [75], the functional non-indifference to one’s own states begins to expand and face outwards, enabling responses to progressively more distant others’ states. Much works remains, to identify policies for informational coupling of subunits that optimize the potentiation of collective intelligence and care. These policies will be as relevant to establishing thriving social structures as to the design of novel general intelligences.

Biology offers many examples of Selves which change on-the-fly—not just during evolutionary time scales, but during the lifetime of the agent. All animals were once a single fertilized egg cell, then became a collection of cells solving problems in anatomical space, and only later developed an emergent centralized Self focused around navigating 3D space of behaviors [76]. Butterflies (and their brains) result from the rapid remodeling of a caterpillar and its very different brain. In addition to these natural examples, recent advances in chimeric and bioengineering technology are enabling an inevitable explosion of diverse forms of life. Hybrots, cyborgs, chimeras, and other combinations of living material, bioengineered components, and software form an astronomically huge option space for possible forms with diverse kinds of bodies and behaviors [1,3,4,33,42,43,45,46,47,77,78,79,80,81]. This requires us to move from the picture of Adam naming a finite set of static animals in the Garden of Eden to frameworks that can handle the full range of life and mind as they can be—all possible sentient beings.

For AI workers, it is important to step back from a neurocentric view of intelligence—life was solving problems long before neurons evolved; thus, a focus on neuromorphic architectures (such as specifically *neural*-network models) is unnecessarily restrictive. All of the main components of neural systems—ion channels, electrical synapses, neurotransmitter machinery, etc., were present long prior to the appearance of brains [82,83]. Indeed, all cells form bioelectrical networks that process information in morphospace in ways familiar to neurosciences (but on a slower time scale) [64]. The emphasis on natural intelligence as fundamentally arising from goal-directed (homeostatic) loops dovetails with key open problems in AI research, with respect to intrinsic motivation [84,85] and goals: how do goals arise in complex systems? How do we predict and manage the goals of collective intelligences (such as robot swarms), ensuring life-positive engineered systems? Evolution is only part of the story, since synthetic living organisms, such as Xenobots—protoorganisms made of frog skin cells [86,87,88], exhibit coherent anatomical, physiological, and behavioral outcomes that have no backstory of selection forces shaping them. The central concept in this new frontier is Care: what do these systems spend energy to try to achieve—what do they care about? What sets the scope and content of their goals?

A focus on Care has two other important implications for AI. First, it suggests that the ability to recognize, manage, and relate to sentient beings is a key capacity and design challenge for AI. Engineered agents are intelligent in part due to their ability to detect intelligence around them, and our development of tools to help understand intelligence in unconventional media around us should be paralleled by advances to enable AI to do the same. Secondly, it provides one answer to the perennial philosophical problem of how to relate to “artificial” beings—a problem which is strongly exacerbated by existing technologies for chimerizing living tissue with engineered replacements. If one’s spouse had a kidney replaced with an engineered organ, does that change anything with respect to the relationship and moral responsibility? Presumably not. How about several organs, and perhaps a part of the brain? All of the brain, as long as the function is preserved? At what point does one go from a “real person with perhaps some irrelevant mechanical augmentations” to a “robot that is just simulating their (perhaps very convincing) social interactions”? It seems clear that such decisions cannot be based on what the putative person is made of or how they came to exist (evolution, embryogenesis, design and factory construction, or some combination of those), and advances in machine learning suggest that they cannot be based on performance in specific problem areas either (IQ tests). What can they be based on? One suggestion is that they can be based on Care. What we should be looking for, in terms of gauging what kind of relationship we can have with, and moral duty we need to exert toward, any being is the degree of Care they can exhibit, either at present or as a latent potential, with respect to the other beings around them.

Existing life forms reveal a continuum of cognitive capacities reaching all the way from our unicellular ancestors to modern advanced animals, and these can be compared based on the shape of their cognitive light cone—the shape and size (in a space-time diagram) of the most grandiose goals toward which they are capable of working (Figure 2). The area inside a system’s cognitive boundaries is the area of Concern—the number and types of things that this system can Care about, in the sense of practical Concern (willing and able to spend energy to monitor and control). There are of course major transitions in evolution [89,90], corresponding to advances in the types of goals a given system can work towards. These have been discussed elsewhere [24]. One really crucial transition is unique, forming what Hofstadter would call a Strange Loop: rather than committing to increasingly more sophisticated goals, one can commit to the meta *goal of increasing one’s goals*. In Buddhist traditions, this intentional expansion of one’s cognitive boundary (and thus the area of one’s concern) finds expression in what is known as the Bodhisattva vow.

## 5. No-Self in Buddhism and Bodhisattva Intelligence

The past decades saw a development of interdisciplinary interest in synthetic modes of knowledge that integrate Buddhist philosophy with contemporary Western philosophy of mind and cognitive science [91]. In fact, the developing domain of contemplative science today has made attempts at developing novel empirical and theoretical language and tools in order to better understand plasticity of mind and brain [92] and to integrate contemplative techniques (such as meditation) into the models of consciousness and cognition [93].

We propose that a number of core Buddhist concepts (in particular the constructed nature of selfhood/no-self, *duḥkha*, universal loving care, and the Bodhisattva) can be profitably used to challenge and to enrich the current work in diverse intelligences, including novel approaches to AI and to biology. In contemporary cognitive science, the mind is sometimes understood as *enactive* [94,95,96], in that the boundaries between cognition, a form of embodiment, and a being’s relationship with the world are understood as pragmatic and non-reducible [97,98]. Similarly, contemporary proposals for the constructed, transient and contextual nature of human selfhood [99] and for its inextricably social/cultural orientation [100] are delineating a direction for understanding forms of life and intelligences that are relational, transient and have malleable boundaries (here understood as cognitive light cones). These fundamental shifts in perspective are particularly suitable for integrating Buddhist and enactive approaches to AI [101]. In the following sections, we will present core Buddhist concepts of selfhood and the Bodhisattva idea to further develop our proposal that intelligence can be understood in terms of Care and the remedying of stress. Our discussion can, in this regard, be seen as resonant with the enactivist tradition, which describes selves as precarious centers of concern [102], as patterned variations [103] of different forms of experienced selfhood, ranging from the notions of minimal self [104] to embodied, affective [98,105], and socially extended/participatory [106,107,108] forms of situated selfhood. What is common in many flavors of enactive and situated cognition models is the emphasis on the dynamic and creative nature of what it means to have or to be a form of self. Importantly, the recent work on participatory and social forms of selfhood, sometimes referred to as the “interactive turn” [109] in enactive cognitive science, stresses the situated nature of cognition and subjectivity within the social worlds and thus invites us to think specifically about ethical or prosocial forms of constitution of self. While our work is inspired by and is respectful of the enactivist tradition, in this paper we are exploring the translational and generative potential of thinking through the idea of Intelligence as Care in resonance with current concepts in biology, the Bodhisattva vow, and developments in AI. Our proposal that Selves be understood as processual cognitive systems and models thereof bears a natural kinship with enactivist thought as developed and evolved over the past three decades [97,98,110,111], and can thus also be discussed and critiqued in the light of phenomenological notions of self-awareness, minimal self, etc. [112,113,114,115,116]. We acknowledge the resonance with contemporary approaches that see the self as enactive, embodied, extended, affective, situated, and interactive, and we hope our work can anchor future conversations with these rich traditions.

According to traditional Buddhist scriptural analyses, the recognition that there is no singular and enduring individual that must survive and prevail serves to undermine self-seeking action at the expense of others and their environment. Therefore, the evolving of intelligence that is aware of no-self—or if we want, intelligence that is *no-self-aware*—is also held to be intrinsically wholesome and associated with concern for the happiness and well-being of others. This claim—that simply understanding the irreality of enduring, singular agents can be a catalyst for ethically informed intelligence—is especially noticeable in Great Vehicle (Skt. *Mahāyāna*) currents of Buddhist view and practice that develop the idea of the Bodhisattva [26,27,117]. Traditionally conceived as an agent that has accepted responsibility for the flourishing of all sentient beings throughout time and space, a Bodhisattva pursues the attainment of “omniscience” (defined as unmediated and simultaneous knowledge of all things throughout space and time, both as they are and as they may seem to cognizing subjects) in order to protect and provide for all beings, and to assist them in achieving their own ultimate potential [118,119]. In this way, understanding of the drive of a Bodhisattva is two-fold: as affectionate care (concerned with sentient beings) and as insight into things as they are and as they appear (associated with the pursuit of omniscience). These two drives, care and insight, are seen as standing in a dynamic relationship and are not separate in essence. Hence, as a model of intelligence, the Bodhisattva principle may be subsumed under the slogan, “intelligence as care”.

Such emphasis on care contrasts to some extent with an understanding of intelligence as, first and foremost, the ability to control causal chains that lead to the achievement of predefined goals. Rather, the “intelligence as care” proposal can be seen as highlighting an element of spontaneity that emerges in the light of the co-constitution of object, agent, and action. According to general Buddhist analysis, the individual that may be assumed to exist as a singular, enduring, and controlling self is mere appearance devoid of causal efficacy, and thus epiphenomenal [68]. In the case of a Bodhisattva, this understanding is carried forward so as to encompass a critique of the apparent foundations of cognition: object, agent, and action. Since the constituents of this triad can neither be determined as distinct from one another, nor identical, both the epistemic and the ontological status of all versions of this triple framework are seen to be on the same level as dream images, mirages, and other such traditional examples of illusion. Thus, recognizing the interdependent and constructed nature of object, agent, and action means recognizing their “emptiness” (Skt. *śūnyatā*), and since “recognized,” “recognizer,” and “act of recognition” are also within the purview of comprehensive deconstruction, the “recognition” in question is often qualified as being of a nondual nature.

According to the Bodhisattva model of intelligence, such deconstruction of the apparent foundations of cognition elicits a transformation of both the scope and acuity of the cognitive system that performs it. As caring intelligences, Bodhisattvas are potentially described as embodying an enlarged field of affordances achieved through the understanding that selves are illusory. Thus, subsequent to the deconstructive insight proper, the appearances of a world inhabited by selves resume. However, at this point the Bodhisattva cognitive system is no longer constrained by the perception that one single self—i.e., its *own* self—requires special and sustained attention. Instead, Bodhisattva cognitive processes are now said to engage with spontaneous care for *all* apparent individuals. Thus, an immediate takeaway from non-dual insight is said to be the perception that oneself and all others are ultimately of the same identity.

In the traditional literature, beings that are not deeply informed by insight into no-self are referred to as “ordinary,” and such non-bodhisattva cognitive systems thus buy into the apparent yet epiphenomenal nature of subjective identity. From the perspective of a mind that in this way reifies personal selfhood, the very sense of being a subject of experience and a controlling agent of actions naturally and unquestionably implies that one is thus also someone who should receive special care and deserves to flourish far beyond the status quo. However, for a Bodhisattva who emerges from the understanding that object, agent, and action are interdependent constructs, this perception of a worthy and deserving self is now said to accompany the perception of any and all sentient individuals—with the same force and naturalness that were previously reserved for the perception of one’s own self. All are in this way seen as equally attractive and naturally worthy of care.

Based on the above contours of the Bodhisattva idea, we shall next seek to analyze this concept of intelligent life with the help of the cognitive light cone framework described earlier (Figure 2 and Figure 3). Let us again note that this model of cognitive life avoids appeal to the notion of a singular, enduring, and controlling self—in other words, the very self that Buddhist perspectives generally agree is unfindable [68]. Instead, according to the light cone model, selves are conceived as evolving s systems and the functional structure of such systems corresponds with the scale of their goals; as such, they shrink, grow, and change over time, as the scope of the system’s goals changes. Such correspondence between goals and agent structure aligns well with a Buddhist understanding of intention as a type of *karman*, or “action,” understood as a world-constructing force [67,120]. As we have also noted earlier, cognitive systems emerge according to this formalism from a hypothesized drive to reduce stress—the difference between current conditions and life-optimal conditions. This definition of the fundamental drive that sustains cognitive evolution in turn resonates with the description of the Bodhisattva’s care and insight drives [26,27]. As we noted above, “stress”, understood as the tension between the present and the optimal state, is an apt translation of the Sanskrit *duḥkha* (otherwise often rendered as “suffering”), which a Bodhisattva’s care seeks to alleviate. Thus, operating through its two-fold (viz. care and insight) drive to reduce stress, the Bodhisattva cognitive system can conveniently be conceived as evolving naturally, driven by basically the same concerns as other forms of life. Furthermore, as a Bodhisattva system evolves and develops agility, its capacity for networking and integration is described as becoming radically enhanced and increasingly spontaneous. This capacity for integration into increasingly comprehensive intelligent systems is described as a hallmark of the cognitive processes that emerge on the “Bodhisattva path” [26,27].

## 6. Bodhisattva Vow and Bodhisattva Path in Relation to the Light Cone Model of Cognitive Boundary

In this way, salient features of light cone formalism align well with traditional features ascribed to Bodhisattva cognition, so an attempt at delineating the latter in terms of the former seems both possible and potentially illuminating. How, then, would the cognitive light cone of a Bodhisattva system have to be drawn? In order to address this question, it is helpful to begin with the beginning of the Bodhisattva path, which traditionally is formulated in terms of a comprehensive commitment. Motivated by loving care for *all* sentient beings, an emerging Bodhisattva vows to achieve omniscience in order to help and provide for all the infinitely many forms and instances of sentient life. When for the first time this “mind of awakening” (Skt. *bodhicitta*) is brought forth with a commitment toward its continuous cultivation and ultimate fulfillment [118,121], then that constitutes the first step on the Bodhisattva path. Based on this commitment, the newly emergent Bodhisattva will also gradually cultivate the deconstructive insight that, as mentioned above, is ascribed a capacity to fundamentally transform the perceptions and abilities of its cognitive agent [26,27,117].

If we seek to analyze the consequences of adopting this Bodhisattva commitment according to the light-cone model of intelligent life, we will notice that the respective structures prior and subsequent to the Bodhisattva pledge will have to be drawn in radically different ways. If, for example, we imagine a human being about to adopt the Bodhisattva perspective, their sphere of explicit concern before making this defining commitment may be more or less expansive by human standards. Indeed, we might imagine that they have explored distant galaxies with a view to future space travel, many generations from now, or that they have developed programs with intended applicability across our globe’s ecosystems. Yet even if their spheres of measurement and activity up to this point have in this way been extraordinarily vast, we will still notice a striking difference in the functional boundary that emerges subsequent to their adoption of the vow: a difference that ensues simply from the way that the vow is formulated. By definition, the Bodhisattva perspective entails active concern across an infinite range, both spatially and in time. In short, wherever the endless myriads of beings may find themselves, the Bodhisattva forms the intention to go there and effect positive change. Implicitly, this programmatic intention thus also contains an open-ended pledge to comprehend the past, because the ability to skillfully influence events in the present and future can be seen to involve knowledge of past states of affairs. Thus, by *simply committing* to the Bodhisattva stance and practices, the sphere of measurement and activity of the cognitive system that makes the commitment has gone from finite to infinite, and so the cone structure that otherwise is applicable to all forms of cognizant life has in this sense been transcended.

**Figure 4:**
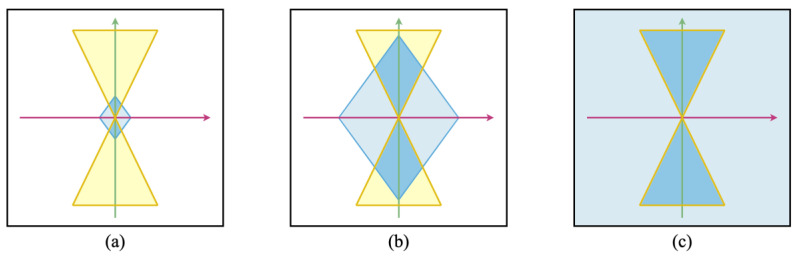
Care boundaries and physical light cones. Legend: Illustration of Care light cones (blue) and physical light cones (yellow). These illustrative plots of light cones represent Care, goals and inspirations in space (horizontal axis) and time (vertical axis) for different agents, represented by (**a**) a tick, (**b**) a human, and (**c**) an agent that has taken the Bodhisattva vow, for that specific agent at some specific time. The plots respond to similar conventions as Figure 4. The limited Care cones depicted in (**a**,**b**) contrast with (**c**), where the field of committed concern has become infinite (as shown by the pervasive blue tone).

**Figure 5:**
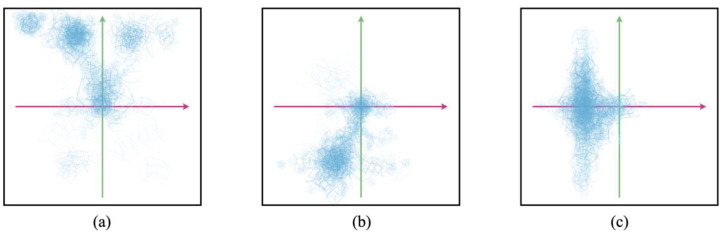
Sketches for three archetypes of Care light cones (CLC), exemplifying the complex aspect of light cones for agents in the world. Legend: While the diamond or “spinning top” shapes of CLC depicted above are simplifications, these sketches show examples of the way an agent’s Care capacity may be depicted in a more nuanced fashion. Each CLC presents a snapshot of the probability distribution of Care in a given agent with a stereotypical profile. In (**a**), the agent is primarily concerned about a selection of complex future scenarios, and this agent is hence dubbed “the futurist.” “The historian” depicted in (**b**) is primarily involved in exploring complexities of the past. Finally, “the chauvinist” represented in (**c**) relies on a one-sided narrative about a deep past to resolve most matters concerning the future. This illustrates how the CLC may for example come in various shapes and densities, and be composed of complex, disconnected point clouds.

Seeking to indicate infinity on both the spatial and temporal axes, we might now instead see the Bodhisattva cognitive system’s computational surface represented by an all-encompassing sphere that accommodates all instances of life within it. On the other hand, a human cognitive system that adopts the Bodhisattva pledge—perhaps through a formalized ritual—is obviously not likely to notice any erasure or infinite extension of its functional boundaries simply due to participation in the vow-bestowing ceremony. Indeed, the dynamics of such a cognitive system will in many ways unfold just as before, based on characteristically human circumstances and conditions. Nonetheless, if the expressed contents of the vow have been registered, the system’s prior functional boundary will now have become open-ended in space and time. Taking into account both the largely continuous character of the human cognitive system and the all-encompassing character of the Bodhisattva sphere of engagement, we may then represent the cognitive system of a newly emergent Bodhisattva in human form by placing a physical light cone (PLC) within an all-encompassing Care light cone (CLC) sphere of infinite commitment (Figure 4). We note that the limited PLC and the infinite CLC are equally required for a representation of our “newborn” Bodhisattva: if the CLC were not coextensive with the field of possibilities we would simply be looking at an instance of human cognition, and without the confining PLC structure we would be concerned with pure perfection, transcendent of context. CLC volumes may come with more complex shapes than the diamond or “spinning top” presented above. Figure 5 illustrates more nuanced cone profiles by showing three arbitrary CLC archetypes. Each profile presents a snapshot of the probability distribution of Care for an agent with a distinct stereotypical profile–“the futurist”, “the historian”, and “the chauvinist”–depicting how the CLC may take various shapes and densities, and be composed of complex, disconnected point clouds.

## 7. Intelligence as Care

While, as noted earlier, the Bodhisattva concept lends itself well to representation according to light cone formalism, we can now further conclude that if we go by that framework, the features of a Bodhisattva system stand out as dramatically distinct from those of other known or putative forms of life. Such dramatic differences would perhaps be expected if we had been seeking to represent the features of highly evolved Bodhisattva states and their associated features of super intelligence, as they appear in traditional accounts. Yet what we just drew represents the cognitive system of a Bodhisattva on the most basic, entry level of the path: a system that for the first time adopts the so-called Bodhisattva vow. As we recall, this vow is formulated as a commitment to cultivating “knowledge of all things” in order to help and provide for all beings.

The fact that tradition speaks of this initial, explicit expression of universal care as “the root of omniscience” is noteworthy, and we may ask ourselves why pledging to be, as it were, infinitely good should be of such pivotal epistemological consequence. Yet if we accept the framework of the cognitive light cone as our model for the emergence and integration of intelligences, it is clear that the formulation of infinite concern and responsibility establishes an immediate link to open-ended, and thus infinite, intelligence. In other words, commitment to the Bodhisattva codex formally renders care limitless in time and space, and according to the light cone schema, care translates into intelligence.

Now, can it similarly be said that the pursuit of intelligence, or knowledge, entails an expansion of care? In assessing this question, we may again employ the same model, this time to an agent that formulates a commitment purely in terms of pledging to know “everything there is to know throughout time and space.” The functional structure of this agent will, it seems, be less impressive than that of a classic Bodhisattva agent where the primary impulse is care. The light cone demarcates the spatiotemporal sphere that an agent “can measure and attempt to modify”. However, compared to care, which drives both measurement and modification, the pursuit of knowledge alone lacks any explicit drive toward modification. This might, in turn, suggest that a quest for “knowledge for its own sake” becomes burdened by a potentially self-defeating passivity. Instead, we might further hypothesize that care is required to engender the dynamics that enable truly revolutionary cognitive developments, such as those leading to superintelligence, or to artificial general intelligence.

To further accentuate the features of the type of intelligence that is associated with the Bodhisattva vow, we may here for comparison consider the frame of mind of someone who vows “I shall subjugate everyone in time and space for my own pleasure”. At first glance, we may in these two cases notice a similar sense of universality and infinity, but a closer look reveals marked differences. In the case of the vow to subjugate for personal enjoyment, the universal commitment is directed toward the fulfilment of the agent’s individual version of what should/must be the case. Personal needs are intrinsically limited. Even though greed may feel infinite, once we begin to specify what we want, our needs become rather limited and predictable, because they largely correspond with our understanding of who and what we are. For example, despite all our arguments and seeming differences, in the end we humans all have rather similar desires with respect to pleasure, wealth, health, etc. So despite the apparent grand scale (wanting to embrace all of time and space), the drive toward the fulfilment of “*all my personal wishes*” becomes quite trivial—just like that of any other Self/sentient being—when compared to the care drive of a Bodhisattva. The scope of the Bodhisattva’s sphere of measurement and modification is not just seemingly infinite, but actually so, because a Bodhisattva’s scope and mode of engagement are not defined by the intrinsically limiting frame of one individual mind. Instead, it is shaped and driven by the infinity of living beings, constituting infinitely diverse instances of needs and desires in time and space. The Bodhisattva promises to know all of those needs, and respond creatively and benevolently to them. In comparison, even if we think, “I shall turn all universes into nothing so that I may be happy!” or “I shall make everything in time and space my personal property!,” the span and intensity of the cognitive sphere that is instantiated and sought to be measured and modified by such wishes seem very narrow and dull when compared to the Bodhisattva’s wholly other-directed drive (“Whatever may make them flourish, let them have it!). The paths and end states of the wish for universal destruction or universal possession are easy to conceive of when compared to the Bodhisattva’s endless path of endless discoveries.

Similarly, what might we learn by juxtaposing the Bodhisattva ideal with the mind of Māra, who at times is referred to as “the Buddhist devil”? In Buddhist scripture, Māra at times occurs as an evil deity (thus famously trying to subdue and distract Siddhartha on the eve of his ultimate awakening) and at others more like a universal principle of evil and deception (as in the framework of “the four māras”) that stands opposed to awakening [122]. The māra principle can be seen to have analogs in physics, which suggests that this is a concept that operates “all the way down” as limitations on the ability of agents to know, predict, and engineer. “Four māras” could, for example, be enumerated as entropy, inertia, Gödel uncertainty, and finite speed of light (viz. the limitations of special relativity). These work hard to keep us all down, and each is a background “force” that opposes all efforts to do good things. Movement, complexification, etc., are all resisted all the time, appearing as an impersonal feature of the universe that is constantly trying to undermine whatever one may want to do. Just as particles have nano-goals—the light beam wants to get to its target by the path that uses up the least energy and so is subject to the influence of “Mara,” in the sense that by moving it will inevitably use up some energy—their very goal-seeking (variational principle) property is the effort of resisting the Mara-like influence.

As a quasi-personal, pervasive principle, Māra has intentions that can be understood as other-directed in a way that is comparable to the Bodhisattva. Māra does not really have any wish “for himself”. Instead, his drive is motivated by a negative formulation of the Bodhisattva pledge: “Wherever there are beings, let me prevent their awakening!” This other-directed sensitivity seems to deliver a light cone that surpasses those associated with the more traditional “selfish” intentions we considered in the paragraph above. As with the Bodhisattva, there is in Māra’s case an open-ended universal drive to both determine and influence each and every actual state of sentient beings throughout space and time. So, rather than yet another subjective mind with personal desires, Māra’s state appears more like pure evil intelligence, beyond personhood. If the Māra drive is in that way pure and all-encompassing evil, the Bodhisattva state is then universal benevolent engagement. How to compare such a pair of intelligences, both other-dependent and other-directed rather than “selfish” in the usual sense? Is one more powerful than the other, or do they scale up the same way in terms of the light cone model? Let us at this point simply note that the Māra drive seems reducible to a wish to maintain the status quo (“sentient beings suffer, and they shall keep doing so!”) whereas the Bodhisattva is committed to infinite transformation. If that is correct, the intelligence of the Bodhisattva’s care should again display decidedly superior features according to the light cone model, because a static wish to maintain what is—even if it is on a universal scale—entails far less measurement and modification than an open-ended pursuit of transformation wherever its potential is encountered.

Presumably, the circular process created by (a) an expanding light cone, (b) increasingly sophisticated intelligence, and hence (c) increasingly demanding challenges can either break down (in regression) or otherwise keep evolving, endlessly. In other words, since a growing light cone and superior intelligence entail an increase in problems that require solution, the expansion of intelligence can in itself never deliver any lasting peace or accomplishment. This seems to fly in the face of standard assumptions with respect to the nature and efficacy of intelligence. Is not the general expectation that the more intelligent we and our environment become, the less stress we will have to encounter? Yet if the above is correct, such an expectation turns out to be rather unrealistic.

The Bodhisattva vow can then be seen as a way of acknowledging, or even welcoming, this forbidding lay of the land (i.e., that there is no end to challenges, no matter how smart we might become) because one gladly (the wow has to be made joyfully…) accepts an endless project of infinite challenge. Traditionally, the ability to do so is associated with recognizing the facts of “no self” as discussed in the opening of this section. Accepting the Bodhisattva vow brings in this way the possibility of expanding intelligence in a steady fashion—free from hesitation, disappointment, fear, and other such factors that can now be seen to arise from misperceptions of the nature of the project. Instead, the vow invites an all-round cultivation of intelligence that does not assume any end state.

Before we begin to draw up specific implications for the way we may want to conceive of and develop AI, let us in concluding this section then note that the Bodhisattva vow can be seen as a method for control that is in alignment with, and informed by, the understanding that singular and enduring control agents do not actually exist. To see that, it is useful to consider what it might be like to have the freedom to control what thought one had next. Would not perfect control of one’s mind imply that one knew exactly what one was going to think, and then subsequently thought it? In that case, whenever a new thought arose, we would, absurdly, be rethinking what we had thought already, or otherwise there would, just as absurdly, have to be an infinite line of prior control modules in place for a single controlled thought to occur. Such consequences suggest that the concept of individual mind control is incoherent. “In control of my mind” (a necessary aspect of the common notion of free will) is logically impossible on the short time scale, but may be coherent on a very long time scale (“I’ve undertaken practices to eventually change the statistical distribution of the kinds of thoughts I will have in the future”). This in turn underscores the importance of long-term strategies, such as a vow to expand cognition.

## 8. [AI] Approaching a Mathematical Representation of the Light Cone Formalism in General and the Bodhisattva Vow in Particular

The points discussed in the previous sections find a strong echo in the field of AI. Below, we attempt to bring concepts from both biology and Buddhism together into the language of AI, and suggest practical ways in which care may enrich each field. This section examines how to frame the complexity of intelligent agents, in their diversity and substrate-dependency. We address how agents may accomplish goals they *care* about by offloading stress and introducing care, with an illustrative example in the game of chess.

Defining intelligence is difficult [28]. For the purposes of this paper, and for the reasons stated in Section 1, the starting point of our working definition of intelligence was the ability to solve problems in some defined space. This definition is necessarily subjective (because it requires the observer to pick a space, and be intelligent enough to detect problem-solving behavior in it), and it is relative—in the eye of the beholder. Problem-solving requires motivation to progress through homeostatic loops of measurement and action (which requires energy expenditure) and is driven by stress loops (system-level propagation of the mismatch between present and optimal conditions). Thus, advanced intelligence is scaled up by increasing the scope of the states that trigger this stress (imperative to act)—it is “the capacity for identifying stress and working toward stress relief”. Further, as presented in parts 1 and 2 of the present paper, we propose, based on knowledge derived from recent advances in understanding the variety of cognitive living systems and from Buddhist ideals of the Bodhisattva path, that understanding of intelligence can be refined and expanded to understand intelligence as care which is scalable and appropriate for a range of cognitive phenomena, including artificial cognitive systems

When seeking to navigate across diverse definitions of intelligence, an easy rule of thumb is complexity. According to any definition, including the homeostatic one above, an intelligent system is expected to offer a simpler solution to a problem than a non-intelligent one. There are multiple measures of complexity [123,124,125] but this latter point should be valid for each such measure. This may mean, in case we consider computational complexity [126], that the entity uses a lesser amount of resources than another entity. In the case of statistical complexity [125], one can find an epsilon machine with fewer nodes to solve the problem.

Once one has reached a tentative framing for intelligence, it is necessary to characterize to some extent its ontological space and structure [127]. The space displays properties of a complex, diverse, and substrate-dependent system. Agents may show their capacity to drive themselves to identify and minimize stress, in a radically different manner based on the environment to which they are subject, so that ordering or classifying them may end up an extremely difficult task. We recognize that any attempt at defining intelligence will end up simplifying, due to the complexity and the loaded character of the term in the literature, especially among different fields and angles from which scholars have tried to frame it. It should be clear to all that the nature of intelligence is extremely diverse. Echoing the discussion in the biology section, we may underline that diversity strongly holds also for artificial systems, and for combinations of artificial and natural systems too. Each intelligence in simulations and AI may solve in a vastly different way the problem of its own existence, and so reduce the stressful gap between what is current and what would be optimal.

Oftentimes, artificial systems are not autonomous of course, and rely on humans maintaining them in order to keep existing. In such cases, one may want to consider the whole system including the maintainer instead of the AI alone. However, in either case, there may be many ways for a given system to implement a solution. For example, a Von Neumann architecture given the task of estimating the value of pi may use two completely different methods: one may be the Monte Carlo method, which generates a large number of random points within a square and counts how many fall in an enclosed circle [128]), or a fast Fourier-Transform-based method, which uses Brent binary splitting together with an efficient cache-handling Hermitian FFT to multiply big integers [129]. Both methods lead to the same result, with slightly different amounts of resources, including a different use of memory and computational power. However, the memory and computational power will differ a lot based on the environment. The same way a human does well on Earth but will struggle in Mars conditions, any artificial agent will behave very differently in a different physical substrate, which may come with a different computational paradigm. Similarly, with quantum computers, some algorithms are asymptotically faster than the fastest possible classical algorithms [130].

## 9. Transfers of Stress

Above, we introduced the notion of stress, understood as an energy function or the distance between a given state and the optimal state, so that reducing stress may act as a driver for a given system to navigate the problem space. A similar situation can be found in AI systems, where optimizing the value of a single parameter is enough to drive a whole algorithm forward in its evolution, whether it is a simulation, an optimization algorithm, or any other learning system in general. Such a parameter is often designated as a reward function, but more generally characterizes a drive for the system to evolve a certain—often intended—direction. For example, in supervised learning, a so-called loss function calculates the distance between the current output of an algorithm and the expected output, and is used to evaluate how accurately an algorithm models a dataset. A drop in the loss function indicates a higher quality prediction, whereas a high value for the loss indicates that the prediction is completely off.

In the case of tasks with complex control sequences or exploration-exploitation tradeoffs [131], another AI technique called reinforcement learning is often used. A reinforcement learning agent is able to perceive and interpret its environment, take actions, and learn through trial and error. The learning takes place as the agent acts, while maximizing as much cumulative reward as possible, which it gains through adopting desired behaviors and minimizing punishment from undesired ones. This notion of reward ultimately drives the behavior of this type of learning system, in which negative reward would be equivalent to the same concept of stress as we mentioned before. These two examples of AI techniques display a strong analogy with the notion of stress introduced earlier in the context of biological systems. Of course, in the case of humans utilizing AI systems, the stress is transferred between the human and the AI. There is a conversion of the stress from human instructions to the AI, so that the AI is driven in the right direction for the final output to be of best use to the handling human. Vice versa, more and more AI systems are designed to express rich feedback to humans so that they may recognize errors or hurdles that may require a change in the way humans run the algorithm.

## 10. Intelligences Working Together/Stress across Levels of Description of Reality

Diverse intelligences may work together, and be organized in layers. In natural systems, one finds numerous levels of organization, from fundamental physical particles at the bottom, up to the biosphere, or beyond the universe. This layered structure has been identified as a layered cake of “reductive levels” [132], “levels of mechanisms” [133], or the “multilevel selection theory” of cooperation in biology [134,135].

The evolution of life on Earth has seen major evolutionary transitions, characterized by individuals which could previously replicate independently, cooperating to form a new, more complex life form [136,137]. For example, archaea and eubacteria formed eukaryotic cells, and cells formed multicellular organisms. A major evolutionary transition involves two steps: first, the formation of a cooperative group; second, transition to a new level of organism, with division of labor, interdependence, and coordination of the parts. This new level constitutes a biological layer [90,138]. This may also translate into a debated framework for studying downward causation [29,139], where higher-level entities or properties may exert causal influence on [140] lower-level ones [54,141].

## 11. Signals That Offload Frustration

One system or level of organization may signal its stress to another one, which may, as a consequence, drive it in such a way that this second system may express a behavior that ends up reducing the stress level in the original system. One may view this phenomenon as a communication system being established between two systems, which allows them to drive each other, sometimes bidirectionally, thus effectively exchanging signals to mutually offload their own frustration. Eventually, for this method to work, a protocol needs to be established between the systems, so that the conditions lead to the right course of actions for the second system to reduce stress in the first system. If successfully established—i.e., if the signal manages to drive system B so as to reduce the stress in system A—a system may be seen as offloading its stress onto another system, as this stress in the first system converts into some structure in the signal sent to the second system, which the second system may then interpret to generate behavior helpful to the first system to achieve certain given objectives. Once established, such protocols may be understood and treated as foundational factors for the creation and evolving of artificial “Bodhisattva agents”. Effectively, the stress acts as a real-time indicator of care—the progress with respect to an objective function for a given system, since minimizing a given parameter may be generalized to optimizing for any well-defined goal.

In keeping with the Bodhisattva model, let us notice that this phenomenon may occur between levels of organization as well, with one layer signaling its stress to the next level of organization. Although single bacterial cells may be driven by a level of stress connected to local sugar levels, the tissues collectively formed by those cells may be concerned with evolving into the right shape and size instead.

## 12. Goals in Learning Systems

In AI, goals are what allow one to explicitly drive a system’s learning. Every AI technology has a mathematically well-defined goal, often a labeled dataset, but sometimes a different paradigm. In supervised learning—which are the most commonly used machine learning techniques—a goal is defined by choosing a training set with determined labels. For example, if one wanted to identify whether a picture contains a cat, the training set may be a set of pictures each labeled as containing a cat or not. The learning system will then be trained to output whether an image contains a cat or not, by being rewarded when giving the correct response. Most of deep learning, neural networks, decision trees, random forests, and logistic regression are all training based on labeled datasets to define their goal objective. In another family of machine learning techniques known as unsupervised learning, a goal is defined too, although in a slightly different or perhaps more subtle manner. In unsupervised techniques such as clustering (k-means, etc.), the goal is very well-defined, as some mathematical cost function to minimize. An example of cost function may be the Euclidean distance between points within the same cluster, and the opposite of the distance between points not within the same cluster. In reinforcement learning, one would define a function to reward or penalize actions, which also determines a goal for the learning algorithm. Such technology has been used recently with a lot of success to play games such as chess and Go, where a sequence of actions is required to reach a particular goal such as a checkmate. The reward function continuously gives hints along the way to determine the correct learning path.

All these machine learning techniques, among many more, include a well-defined goal. In other classes of algorithms, the goal might be implicitly defined as part of the environment or the simulation in use. In evolutionary computation or genetic algorithms, for example, a population of mathematical functions is constantly evolved and selected so as to perform best on a given problem. The choice of the problem will determine the goal, most often in a “fitness function” that defines the reward, ultimately used to choose the path to learning for the algorithm. At first, these examples may feel somewhat distant from the way we view biological entities, including humans, having goals. However, they have in common that they drive the entity toward their future states. The notion of stress mentioned above should be similar in that respect, since it drives the system in a certain trajectory in time and space. Next, we explain a way to characterize the constraints over the states of agents and their goals, which correspond to two types of light cones, one over physical states, and the second over goals. We argue that the latter is equivalent to care.

To further clarify our description of care light cones, let us consider a simple example using the game of chess, an abstract strategy game where players take turns moving their pieces to achieve the goal of checkmating the opponent’s king. In the Figure 2 diagram, if we were to consider an agent only playing chess, each point of the space would correspond to a certain board position. This board position would be possible or not to reach (in the future) or having been reached (in the past) from the current position according to the rules of chess, based on whether it is located inside or outside the physical light cone. The points in the blue care light cone may for example be a board position which contains a checkmate. Points too distant in space or time to be affected by the agent might be for example a chess player aiming at performing a ladder checkmate, while the pieces necessary for it, rooks and queen, are all missing from the board. Some states in the physical light cone of possible moves in a game might be beyond the light cone of care because the player is incapable of reading far enough in advance or into the past, but they still remain achievable through a certain sequence of moves respecting the rules of chess. Our chess player will not calculate or consider moves that are out of their care volume. A hypothetic perfect player in a solved game, who would see all possibilities in the game, would have their care light cone extend infinitely over the whole space of possible boards, while an empty care light cone might mean the agency of the entity ceases to exist.

Earlier, we also mentioned that artificial intelligences may emerge or evolve in various substrates, adapting and constrained by them, which in turn creates different types and degrees of intelligences. Diversity in types of “intelligences” in our example of chess would lie in the fact that chess players display various styles in playing the game, in terms of style or paths to victory. The substrate is typically a human brain, but instead of playing the game themselves, one may write an algorithm to play the game in their stead, which would typically run on a machine and possess various types of strategies and various degrees of success against certain opponents, defined both by its code and the machinery running it. Diversity in terms of chess skill, if not evident, is exemplified in the light of the game not always needing to be transitive [142], i.e., for any triplet of players A, B, and C, A being consistently stronger than B and B being stronger than C does not necessarily mean that A will be consistently stronger than C. An anecdotal example is a series of matches of Tal vs. Spassky. The years around Tal’s winning the World Championship, 1954–1965, he scored 2-9 in wins against Spassky. One of the wins came from a lost position, which could have made it a 1-10. Spassky’s skill level peaked after that period, but Tal still went 5-0 against him after 1965. The same way chess is non-transitive for human players striving for victory determined by the rules of the game, so are other environments where agents must strive to achieve specific goals under different rules. This goes to illustrate how agents may display a high diversity of paths to achieve their goals and reduce their levels of stress. Chess is a typical domain where hybrid play (two or more players playing as one) is commonly found, either as a combination of several human players, several AI engines, or a combination of both. This may yield a simple case study of a system where agents with mixed stresses may stretch their respective care in such a way that the system as a whole performs better at the game from their interaction, and relates to the impending enrichment of our world with a broad diversity of evolved, designed, and hybrid agents [4].

## 13. Ethics

Novel technologies are exploiting the plasticity and interoperability of life to create novel living beings, such as hybrids, chimeras, cyborgs, brain-computer interfaces, etc., resulting from living tissue tightly integrated with designed inorganic interfaces [39,40], biological robots [42,43,44], neuroprosthetics [143,144,145,146,147,148], and hybrots consisting of living brain tissue instrumentized to control artificial new bodies [1,43,45,46,47,48,49]. Our future will involve a highly diverse space of novel beings in every possible combination of evolved cellular material, designed engineered components, and software. How do we know what we should expect from intelligences in unconventional embodiments? How do we relate to them, and what do we owe them, in a moral sense? Current distinctions that rely on the origin (evolved or designed) or composition (biological vs. technological) of agents will not survive the next couple of decades. These novel, unconventional beings will not resemble any other familiar touchstone in the evolutionary phylogenetic stream. In contrast to Western philosophies rooted in an essentialism of the Garden of Eden, where Adam named a standard set of animals, Buddhist concern for “all sentient beings” is suited to the astronomically large option space of possible beings.

In the absence of common markers (such as brain size), we must establish a rubric within which to compare truly diverse intelligences and set rational policies for proportional moral relationships between very different beings. One such is the measurement of the area of Concern: we can gauge the degree of each being’s possible radius of compassion, create only beings with large, outward-facing compassion capacity, and at the same time enlarge our own agency and intelligence by acting on the Bodhisattva vow.

○ How do we pick our goals? Try to dissolve goals? That dissolves the Self. Or enlarge goals to improve lives of other beings? Bodhisattva enlargement.
○ How to live a good life when binary notions of real and false are increasingly called into question and we are called upon to skillfully occupy the space between them?
○ What does it mean to merge with other selves? Is that something we want?

## 14. Conclusions

Stress drives agents towards homeostatic goals—a concept central to Buddhism with its teaching of existence as dissatisfaction, *duḥkha*. Expanding one’s space of possible goals to face outwards, exhibiting compassion toward other agents’ goals, potentiates the increase of intelligence and thus the potential to identify better, more global solutions. In this scheme, compassion (in the sense of practical concern) and intelligence are tightly linked because the driving definition of an active agent is the bundle of processes that expend energy toward system-level goals. The scale and content of what a given agent measures, prefers, and seeks to implement define its cognitive sophistication. All beings (including humans, synthetic organisms, and engineered AI) can expand their cognitive boundary, working on the meta-goal of enlarging and turning outward (toward the struggle of other sentient beings) their capacity to Care. The Bodhisattva vow is an example of how this can be done and can be viewed through the lens of biology, cognitive science, AI, and Buddhism, which are surprisingly coherent in their emphasis on Care as a central invariant across diverse embodiments.

Here, we have considered a framework for defining intelligence in terms of stress reduction or stress transfer. We have discussed how this cognitive light cone model can be used for comparing instances of evolving intelligence in terms of their ability to identify stress and their alleviation at increasingly complex scales, and we have paid special attention to the way a Buddhist account of increasing intelligence through increasing care, specifically as associated with the Bodhisattva vow, may play out along the lines of this model. If we extend this model of intelligence into AI contexts, we might suggest that a radical expansion of the definition of goals, structured around the principle of care for the alleviation of stress, would be required for growth processes that might ultimately lead to systems of artificial general intelligence. Above all, we have identified Care as a central invariant concept across biology, AI, and Buddhism, to capture the motivation, stress, and goal-directedness of agents. This concept may offer some directions of improvement for both natural and artificial intelligence, by committing to expanding the cognitive boundary or the light cone introduced above.

The concept of a Bodhisattva, given its infinite goals and infinite care, provides us with a roadmap towards hyperintelligence, where the scope of goals and their quality/impact are constantly improving. The Bodhisattva vow is a critical point in the evolutionary or personal continuum of intelligence of any agent because it initiates a positive feedback loop and triggers a “great evolutionary transition” [75,90,149,150] in individuality. Strategies that focus on implementing the Bodhisattva vow are a path for enabling a profound shift from the limited scope of current AIs and their many limitations. Consistent with a central concept of Buddhism—commitment to seemingly unachievable goals—the building of agents capable of undertaking the Bodhisattva vow is a profound challenge. However, progress along this path is as essential for our personal efforts toward personal growth as for the development of synthetic beings that will exert life-positive effects on society and the biosphere. Above all, the concept of Care provides a strong and fundamental link between practical strategies that will enhance engineering capacities and a way to develop a mature system of ethics that will be essential for a future in which highly diverse sentient beings must coexist and thrive together.

## Glossary

It should be noted that the below definitions (especially of terms like Self) are not claimed to be the only possible way to naturalize these concepts, but are proposed as parts of one conceptual framework to be evaluated.

Bodhisattva	An evolving, transformative system of other-directed Care. Bodhisattva agents are traditionally described as committed to the pursuit of cognitive perfection (“awakening,” *bodhi*) for the purpose of assisting all other beings in reducing their stress and achieving optimal circumstances.
Bodhisattva vow	The Bodhisattva’s commitment to infinite Care is formalized in the Bodhisattva vow. Taking the vow initiates the Bodhisattva evolutionary processes and sustains the evolving agent.
Care	Concern for the alleviation of stress. Propelled by the perception of stress, an agent’s Care may focus on the goal of stress relief within its own system but may also be altruistically directed at systems and Selves that may be perceived or otherwise classified as external. In either case, Care finds the status quo dissatisfactory and intends to effectuate change. Care for stress reduction may be spontaneous but can also be deliberately cultivated in relation to the goals of perceived optimal circumstances.
Cooperation	The behavior by one or several individuals with a benefit to another individual, which may be reciprocal or not. In addition, activity of multiple individuals toward a common goal or outcome, within or across scales of organization.
*Duḥkha*	A pervasive fabric of suffering, change, and dependency that is encountered in some form by all self-identifying agents, driving them restlessly forward in an endless pursuit of perceived superior states. In Buddhist thought, *duḥkha* remains in this way a punishing but inevitable general existential condition, and yet the understanding of the very nature of *duḥkha* is regarded as the gateway to liberation. The wish for one’s own private liberation from *duḥkha* is considered anathema for a Bodhisattva. In the Bodhisattva context, the perception of *duḥkha* throughout the world is instead understood as a driver of universal care, and so also of ultimately infinite intelligence.
Evolutionary transition	When a set of individuals cooperate to form a new, more complex life form—such as when archaea and eubacteria formed eukaryotic cells, or cells formed multicellular organisms—an evolutionary transition may take place, with the formation of a cooperative group, followed by the transition to a new level of organism characterized by division of labor, interdependence, and coordination of its parts.
Light cone	In the representation of light cones, the two diagonal lines represent the two extrema in terms of physical change of the system state, while the horizontal line indicates the present state space. Anything outside of the cones cannot be reached from the present state in the future, nor can they influence the present state from the past. For the care light cone (CLC) that is in focus in this paper, the cone represents the boundaries of cognitive ability of agents, characterized in the agent’s goal space, focusing on their cared-for states at a given point in time rather than the states they are actually taking.
Intelligence	The ability to identify stress and the means for its alleviation. In this way, intelligence is the functional ability to solve problems by navigating various action spaces. Intelligence has no privileged physical implementations, anatomical structures, or time scales. Intelligence is a spectrum, beginning with very simple homeostatic loops exhibiting metabolic goals focused on continued existence. Advanced intelligence exploits additional levels of self-modeling which enable multiple levels of virtual modeling of the Self and its outside world (counterfactual thought), anxiety, and creativity (identifying opportunities, not only solving problems existing right now).
Problem space	A mathematical structure imposed on a system by an observer (or the system itself) which allows behavior to be represented as navigating a space of possible states, where some are preferred over others. The ability to find preferred states by taking action is often seen as problem-solving (reducing stress induced by distance from preferred regions), which can be accomplished with various degrees of competency depending on the agent’s sophistication and prior experience. Problem spaces include familiar 3D space which animals navigate via movement, as well as other spaces such as metabolic space, physiological space, transcriptional space, and anatomical morphospace, all of which offer opportunities to reach specific goals, as well as barriers such as local optima traps, complex topology, and inertia.
Self/agent	A temporary, coherent, dynamically changing autopoietic system emerging within a set of integrated parts that (1) serves as the functional owner of associations, memories, and preferences, (2) is the subject of Care, stress, and intelligence, and (3) acts to accomplish goals in specific problem spaces (where those goals belong to the collective and not to any individual subcomponent). Selves are defined by the spatiotemporal scale and nature of the types of goals they can pursue—their “cognitive light cone” [23]. They have functional boundaries and material implementations but are not identical with any specific type of substrate, and can overlap within other Selves at the same, higher- and lower-level Selves. A Self is a theoretical construct, a causal “center of gravity” with respect to closed loops of measurement and action, posited by external systems (such as scientists, engineers, and conspecifics) and by systems themselves (via internal self-models).
Stress	A system-level state which serves as a driver for homeostatic loops operating over a variable that is progressively reduced as activity brings the system closer to its desired region of action space. The spatiotemporal and complexity scale of events that can possibly stress a system is a good indicator of that system’s cognitive sophistication. Stress can arise via discord between external states and the Self’s needs, between sensory stimuli and expectations, and between the goals of multiple subsystems within or across agents. Selves may come to reduce their levels of stress and transfer them between each other in efficient ways, which requires signaling their goals between each other.
